# Is even a probable premenstrual dysphoric disorder diagnosis associated with more severe anxio-depressive symptoms and lower well-being? A preliminary cross-sectional exploratory study

**DOI:** 10.1192/j.eurpsy.2023.1298

**Published:** 2023-07-19

**Authors:** I. Kovács, B. Pataki, B. L. Kis, S. Kálmán

**Affiliations:** 1 Department of Psychiatry, Albert Szent-Györgyi Medical School, University of Szeged, Szeged; 2 Institute of Psychology, Károli Gáspár University of the Reformed Church in Hungary, Budapest; 3 Albert Szent-Györgyi Medical School, University of Szeged; 4 Department of Behavioral Sciences, Albert Szent-Györgyi Medical School, University of Szeged, Szeged, Hungary

## Abstract

**Introduction:**

Premenstrual dysphoric disorder (PMDD) is a newly introduced category in the 5^th^ version of the Diagnostic and Statistical Manual of Mental Disorders (DSM-5) and is highly underdiagnosed worldwide, despite its strong connections to anxiodepressive symptom severity and perceived well-being.

**Objectives:**

Firstly in Hungary, our aim was to: (a)assess whether even a probable PMDD diagnosis is associated with elevated levels of depressive and anxiety symptoms, and decreased perception of well-being on an adult women sample; (b)to evaluate whether women with a probable PMDD diagnosis report greater fluctuation of their affect during the different phases of their menstrual cycle; (c)to examine whether the elevated levels of anxiodepressive symptoms and lower well-being increase the statistical likelihood of having a probable PMDD diagnosis.

**Methods:**

An online test battery was designed to examine probable PMDD diagnosis, severity of anxiodepressive symptoms and well-being. 393 adult women were screened for eligibility in the study (exclusion criteria involved: irregular cycle; use of hormonal contraceptives), from which 112 were included in the final analyses. DSM-5-Based Screening Tool for Women’s Perceptions of Premenstrual Symptoms, Beck’s Depression Inventory, Spielberger’s State Anxiety Inventory, and the WHO-5 Well-Being Index were assessed.

**Results:**

Based on the DSM-5-Based Screening Tool, the sample was divided into 1)women with probable PMDD diagnosis (PMDD group,n=67) and 2)women without probable PMDD (nonPMDD group,n=45). Menstruation cycles were sorted into menstrual, from-menstruation-to-ovulation, early luteal and late luteal phases. The PMDD group exhibited significantly higher depressive (F(1;56,2)=19.394, *p*≤0.001) and anxiety (F(1;35,6)=17.714,*p*≤0.001) symptom severity and lower scores of well-being (F(1;44,3)=4.288,*p*=0.0442) compared to the nonPMDD group regardless of which menstrual cycle they were in. Binomial logistic regression model was used to test whether higher anxiodepressive symptoms and lower scores of well-being increase the likelihood of having PMDD: the model was significant (χ2(2)=27.287, *p*≤0.001), and it explained 29.2% of the variance in PMDD. Elevated levels of anxiety (B=0.058, S.E.=0.022, Waldχ2(1)=7.142, *p*=0.008,OR=1.060) and depressive (B=0.085,S.E.=0.031,Waldχ2(1)=7.480,*p*=0.006,OR=1.089) symptoms increased significantly the likelihood of having a probable PMDD diagnosis.

**Conclusions:**

Women with even a probable PMDD diagnosis exhibited significantly elevated levels of anxiodepressive symptoms and lower scores of well-being regardless of which menstrual phase they were assessed in compared to women without meeting the criteria of the PMDD screening tool. These preliminary results underscore the need for prospective clinical studies of differences in affective symptoms exhibited in PMDD.

**Disclosure of Interest:**

None Declared

